# Inhibition of Src Family Kinases Ameliorates LPS-Induced Acute Kidney Injury and Mitochondrial Dysfunction in Mice

**DOI:** 10.3390/ijms21218246

**Published:** 2020-11-03

**Authors:** Eun Seon Pak, Md Jamal Uddin, Hunjoo Ha

**Affiliations:** Graduate School of Pharmaceutical Sciences, College of Pharmacy, Ewha Womans University, Seoul 03760, Korea; louisa9419@gmail.com (E.S.P.); hasan800920@gmail.com (M.J.U.)

**Keywords:** acute kidney injury, inflammation, mitochondrial biogenesis, oxidative stress, Src family kinases inhibitor

## Abstract

Acute kidney injury (AKI), a critical syndrome characterized by a rapid decrease of kidney function, is a global health problem. Src family kinases (SFK) are proto-oncogenes that regulate diverse biological functions including mitochondrial function. Since mitochondrial dysfunction plays an important role in the development of AKI, and since unbalanced SFK activity causes mitochondrial dysfunction, the present study examined the role of SFK in AKI. Lipopolysaccharides (LPS) inhibited mitochondrial biogenesis and upregulated the expression of NGAL, a marker of tubular epithelial cell injury, in mouse proximal tubular epithelial (mProx) cells. These alterations were prevented by PP2, a pan SFK inhibitor. Importantly, PP2 pretreatment significantly ameliorated LPS-induced loss of kidney function and injury including inflammation and oxidative stress. The attenuation of LPS-induced AKI by PP2 was accompanied by the maintenance of mitochondrial biogenesis. LPS upregulated SFK, especially Fyn and Src, in mouse kidney as well as in mProx cells. These data suggest that Fyn and Src kinases are involved in the pathogenesis of LPS-induced AKI, and that inhibition of Fyn and Src kinases may have a potential therapeutic effect, possibly via improving mitochondrial biogenesis.

## 1. Introduction

Acute kidney injury (AKI) is defined by a rapid increase in serum creatinine (≥1.5 times the baseline within 7 days) or a rapid decrease in urine volume (<0.5 mL/kg/h for 6 h) [1]. Numerous studies have shown that AKI is associated with high morbidity and mortality in critically ill patients, and with high healthcare costs [2,3]. In addition, AKI can contribute to the progression of chronic kidney disease (CKD) and end-stage kidney disease (ESKD) [4]. The pathophysiology of AKI is multifactorial and involves inflammation, tubular injury, and vascular damage [5,6,7]. Since many targeted clinical trials for AKI have failed [8,9], discovering the detailed pathogenic mechanism and developing novel therapeutic agents is essential.

The Src family of kinases (SFK), non-receptor tyrosine kinases, is comprised of Fyn, Src, Yes, Lyn, Fgr, Lck, Hck, Blk, and Yrk. All members contribute to many aspects of cellular function including proliferation, survival, adhesion, migration, invasion, and metabolism [10]. SFK are widely expressed in a variety of tissues [11], particularly under pathologic conditions [12].

SFK expressed in the kidney play a role in the pathogenesis of kidney dysfunction. Src and Fyn kinases have been shown to be related to diabetic kidney disease [13,14] and unilateral ureteral obstruction (UUO)-induced tubulointerstitial fibrosis [15,16], respectively. Pharmacological inhibition of SFK using PP2 [13,14,15,16] and SU-6656 [13,14] have been effective in treating CKD in vivo and in vitro. We have recently reviewed the possible role of Fyn in AKI [17]. Although the evidence is limited, one study has shown that an SFK inhibitor, PP1, is effective in attenuating AKI induced by ischemia/reperfusion (I/R) [18]. However, the detailed mechanism of SFK in AKI remains elusive.

Mitochondria are important organelles that supply cellular energy [19]. Both mitochondrial function and biogenesis are associated with their morphology, number, and localization/positioning [20]. Alterations in mitochondrial function or biogenesis play a critical role in various pathological conditions [21]. The kidney is one of the most energy-demanding organs and it is rich in mitochondrial content [22]. Previous studies have demonstrated that mitochondrial dysfunction is a critical contributor to the pathogenesis of AKI [23,24]. The expression of PPARγ coactivator-1α (PGC-1α), a master regulator of mitochondrial biogenesis [25], is negatively associated with kidney injury during AKI [26]. TNF-α reduced PGC-1α expression and mitochondrial oxygen consumption in human proximal tubular epithelial cells, which was reversed by PGC-1α overexpression [26]. Global and proximal tubule specific PGC-1α depletion retarded the recovery from lipopolysaccharides (LPS)-induced kidney injury in mice [26].

Interestingly, SFK regulates mitochondrial tyrosine phosphorylation [27,28] and oxidative phosphorylation (OXPHOS) [29,30]. Src overexpression impairs mitochondrial OXPHOS in HepG cells, which is recovered by PP2 [30]. The role of SFK on mitochondrial biogenesis in AKI has not been reported.

The present study examined whether SFK plays a role in AKI through dysregulation of mitochondria. LPS was used to induce AKI in C57BL/6 mice as well as in mouse proximal tubular epithelial (mProx) cells, the major site of AKI. PP2, a pan SFK inhibitor, was used to determine the involvement of SFK.

## 2. Results

### 2.1. PP2 Ameliorates LPS-Induced Mitochondrial Dysfunction and Tubular Injury in mProx Cells

**Figure 1:**
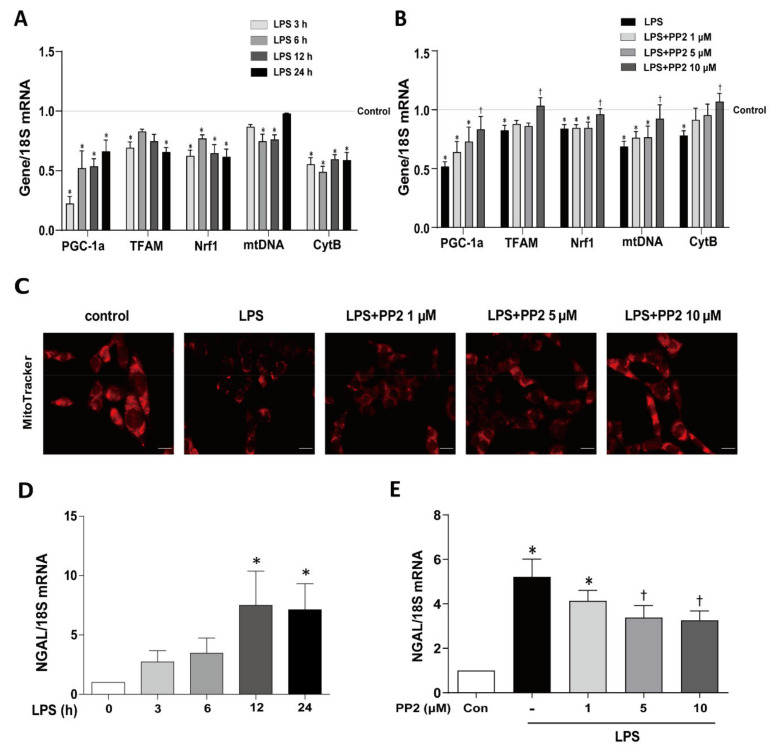
PP2 maintains mitochondrial biogenesis in mProx cells. (**A**,**D**) The mProx cells were stimulated with lipopolysaccharides (LPS) in a time-dependent manner (0, 3, 6, 12, and 24 h). (**A**) The mRNA levels of mitochondrial fitness markers such as PGC-1α, TFAM, Nrf1, mtDNA, and CytB were measured. (**B**,**C**,**E**) The cells were pretreated with PP2 (0, 1, 5, and 10 μM) for 2 h and then stimulated with LPS (100 ng/mL) for 9 h. (**B**) The mRNA levels of PGC-1α, TFAM, Nrf1, mtDNA, and CytB were measured. (**C**) The mitochondria in mProx cells were labeled with MitoTracker staining. (**D**) The mRNA levels of NGAL were measured in mProx cells after LPS (100 ng/mL) stimulation in a time-dependent manner (0, 3, 6, 12, and 24 h). (**E**) The mRNA levels of NGAL were measured. The levels of mRNA expression were measured using real-time RT-PCR analysis and normalized with 18S. Data are presented as mean ±SE, *n* = 4. * *p* < 0.05 vs. control. ^†^ *p* < 0.05 vs. LPS.

We first determined the effect of PP2 on LPS-induced dysregulation of mitochondrial biogenesis in mProx cells. As expected, the mRNA expression of PGC-1α, a master regulator of mitochondrial biogenesis, was decreased by 78% as early as 3 h after LPS exposure and was persistently reduced for up to 24 h (Figure 1A). The mRNA expression of other markers of mitochondrial fitness including TFAM, Nrf1, mtDNA, and CytB were also decreased in response to LPS (Figure 1A). Pretreatment of PP2 at 10 μM effectively restored the LPS-induced suppression of PGC-1α, TFAM, Nrf1, mtDNA, and CytB mRNA expression in the cells (Figure 1B). Mitochondria stained by MitoTracker were also decreased in response to LPS, which was effectively prevented by PP2 in a dose-dependent manner (Figure 1C). LPS upregulated mRNA levels of NGAL, a marker of tubular epithelial cell injury, which was significantly attenuated by PP2 in a dose-dependent manner (Figure 1D,E).

### 2.2. PP2 Improves Kidney Function and Attenuates Kidney Tubular Injury in LPS-Induced AKI

**Figure 2:**
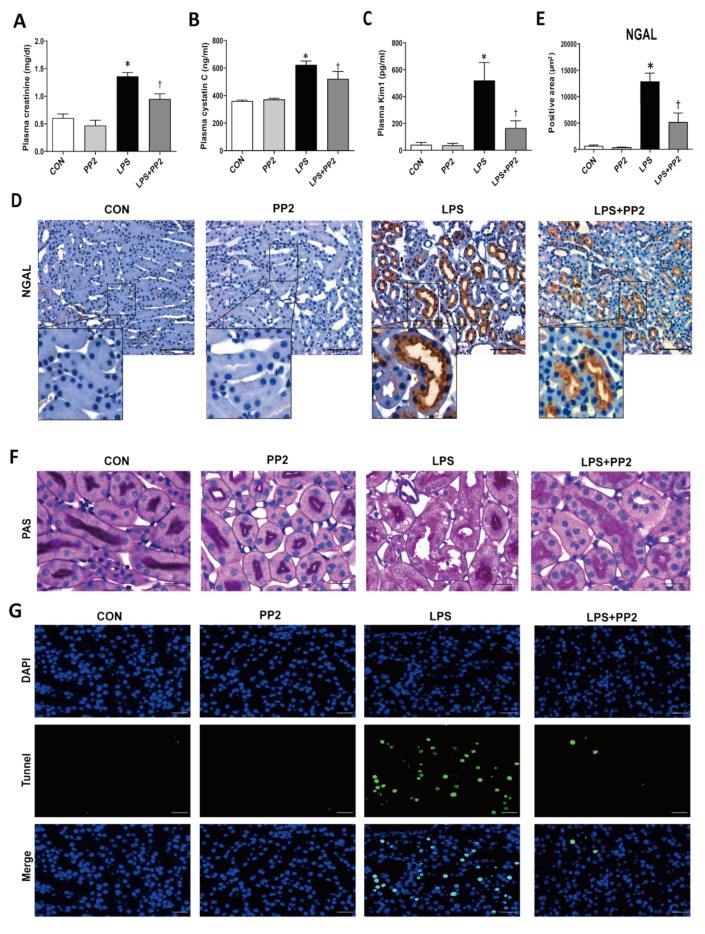
PP2 improves kidney function and attenuates kidney tubular injury in LPS-induced acute kidney injury (AKI) mice. Blood plasma was analyzed for (**A**) plasma creatinine (mg/dL), (**B**) plasma cystatin C (ng/mL), and (**C**) tubular injury marker, plasma Kim1 (pg/mL). Paraffin-embedded kidney sections were stained with (**D**,**E**) NGAL antibody (1:200; original magnification: 200×; scale bar: 100 μm: enlarged images have been shown in inset) and (**F**) PAS staining (original magnification: 630×; scale bar: 20 μm). (**G**) Apoptosis was measured in kidney sections using the terminal deoxynucleotidyl transferase-mediated dUTP nick end-labeling (TUNEL) assay (original magnification: 400×; scale bar: 100 μm). Data are presented as mean ±SE of 6–8 mice. * *p* < 0.05 vs. control, ^†^ *p* < 0.05 vs. LPS.

We next examined the kidney tubular epithelial cell injury in mice after LPS exposure. The expression of NGAL mRNA was increased in the kidneys after LPS exposure for up to 24 h (Figure S2). To examine the effect of PP2 on kidney function and the morphology changes in LPS-induced AKI, we collected blood and kidney samples after 18 h of LPS administration. The kidney injury markers such as plasma creatinine and cystatin C were elevated in response to LPS, which was prevented by PP2 (Figure 2A,B). Then, we determined that the tubular injury as measured by plasma KIM-1 and NGAL immunostaining was also increased in response to LPS, which was prevented by PP2 (Figure 2C–E). PAS staining showed a clear increase in tubular injury in response to LPS, which was ameliorated by PP2 (Figure 2F). Tubular cell apoptosis indicated by TUNEL staining was increased in LPS-induced AKI mice and this was decreased by pretreatment with PP2 (Figure 2G).

### 2.3. PP2 Ameliorates Inflammation in LPS-Induced AKI

**Figure 3:**
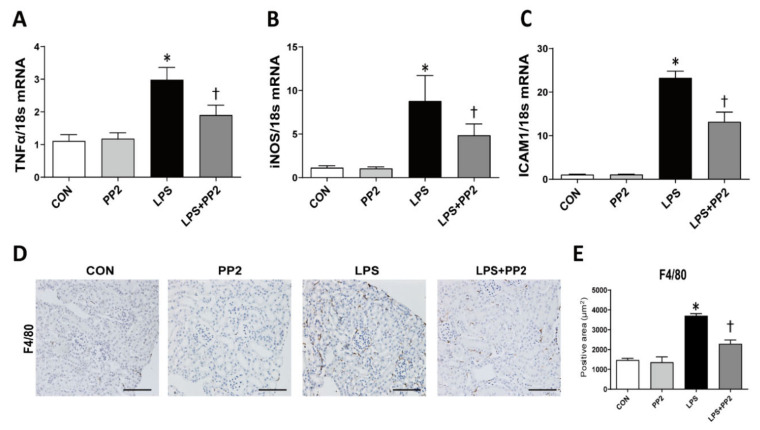
PP2 inhibits kidney inflammation in the LPS-induced AKI mice. (**A**–**C**) The mRNA levels of inflammation markers such as TNF-α, iNOS, and ICAM1 in kidney tissue were measured using real time RT-PCR analysis. The levels of mRNA were normalized with 18S. (**D**,**E**) Paraffin-embedded kidney sections were stained with anti-F4/80 antibody (1:400; original magnification: 200×; scale bar: 100 μm). Data are presented as mean ±SE of 6–8 mice. * *p* < 0.05 vs. control, ^†^ *p* < 0.05 vs. LPS.

Inflammation is critically involved in AKI. In order to determine whether PP2 has an anti-inflammatory effect, we evaluated the expression of proinflammatory cytokines in AKI mice. LPS increased the mRNA expression of tumor necrosis factor-α (TNF-α), inducible nitric oxide synthase (iNOS), and intracellular adhesion molecule-1 (ICAM-1) in AKI mice, which were all alleviated by PP2 (Figure 3A–C). In addition, macrophage infiltration as measured by F4/80 staining was also increased in response to LPS, whereas pretreatment with PP2 decreased these effects (Figure 3D,E).

### 2.4. PP2 Attenuates Oxidative Stress in LPS-Induced AKI

**Figure 4:**
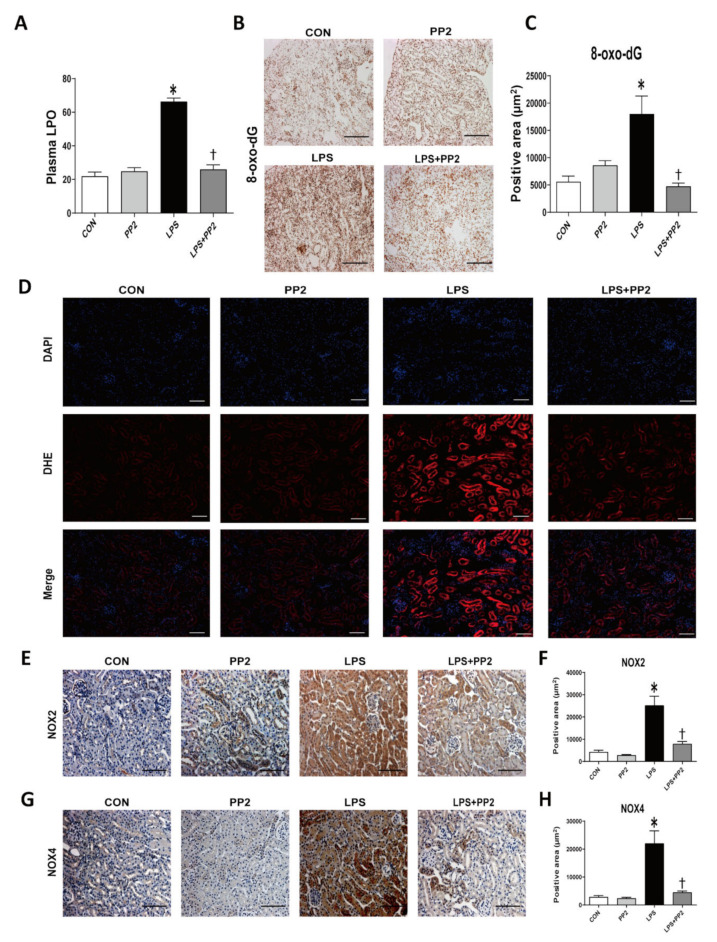
PP2 attenuates kidney oxidative stress in the LPS-induced AKI mice. (**A**) Plasma lipid hydroperoxide (LPO) (μM). (**B**,**C**) Paraffin-embedded kidney sections were stained with 8-oxo-dG (1:200; original magnification: 100×; scale bar: 200 μm) antibody. (**D**) Frozen kidney sections were stained with DHE dye (5 μM for 10 min; original magnification: 100×; scale bar: 50 μm). (**E**–**H**) Paraffin-embedded kidney sections were stained with Nox2 (1:500; original magnification: 200×; scale bar: 100 μm) and Nox4 (1:400; original magnification: 200×; scale bar: 100 μm) antibodies. Data are presented as mean ±SE of 6–8 mice. * *p* < 0.05 vs. control, ^†^ *p* < 0.05 vs. LPS.

We evaluated the state of oxidative stress in AKI mice through the use of the lipid hydroperoxide (LPO) assay in plasma samples and through 8-oxo-dG immunostaining of kidney tissues. LPS increased the LPO concentration and 8-oxo-dG expression, which were all attenuated by PP2 (Figure 4A–C). DHE staining was performed to directly measure the reactive oxygen species (ROS) in the kidney tissue. LPS effectively induced the ROS level, which was reduced by PP2 (Figure 4D). In addition, the expression of Nox2 and Nox4 as measured by immunostaining were increased in response to LPS, and were reduced by PP2 (Figure 4E–H).

### 2.5. SFK, Mainly Fyn and Src, Are Increased in LPS-Induced AKI

**Figure 5:**
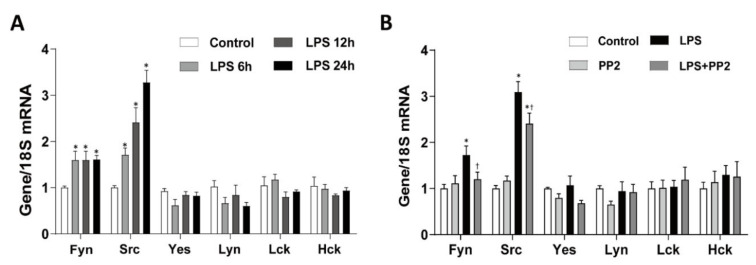
LPS increases Src family kinases (SFK) mRNA expression in AKI mice. (**A**) Six-week-old male C57/BL6 mice were treated with LPS (15 mg/kg) in a time-dependent manner (0, 6, 12, and 24 h). (**B**) Six-week-old male C57/BL6 mice were pretreated with PP2 (2 mg/kg) for 2 h and then administered LPS (15 mg/kg) for 18 h. (**A**,**B**) The levels of SFK mRNA expression were measured using real-time RT-PCR analysis. Levels of mRNA were normalized with 18S. Data are presented as mean ±SE of 6–8 mice. * *p* < 0.05 vs. control, ^†^ *p* < 0.05 vs. LPS.

**Figure 6:**
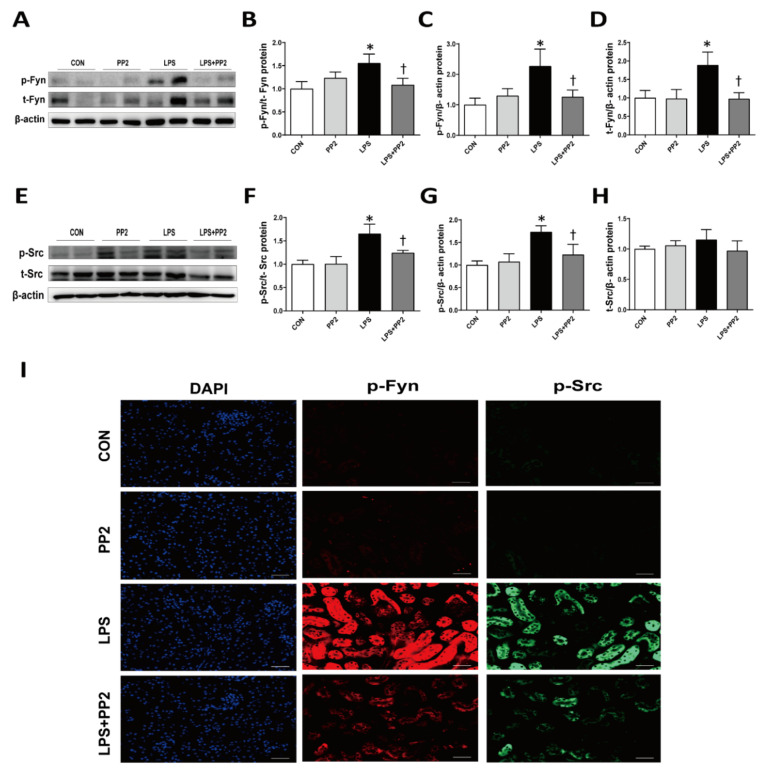
PP2 inhibits Src and Fyn kinase expression in LPS-induced AKI mice. Six-week-old male C57/BL6 mice were pretreated with PP2 (2 mg/kg) for 2 h and then administered LPS (15 mg/kg) for 18 h. (**A**–**H**) Immunoblotting analysis of phosphorylated/total Fyn and Src kinase in the kidney. Levels of proteins were normalized to their respective total protein. (**I**) Kidney sections were subjected for immunofluorescence staining with phospho-Fyn (red) and phospho-Src (green) antibodies and 4’,6-diamidino-2-phenylindole (DAPI; blue). Data are presented as mean ±SE of 6–8 mice. * *p* < 0.05 vs. control, ^†^ *p* < 0.05 vs. LPS.

We examined the levels of SFK in the kidney after LPS injury. Interestingly, mRNA expression of SFK, i.e., Fyn and Src, were increased in the kidneys after LPS exposure for up to 24 h (Figure 5A). Similarly, the expression of Fyn and Src mRNA was increased in the mProx cells in response to LPS (Figure S1). Pretreatment with PP2 decreased the LPS-induced upregulation of Fyn and Src mRNA expression in the kidneys (Figure 5B). In addition, phosphorylation of Fyn (p-Fyn) and Src (p-Src) was upregulated in response to LPS, which was blocked by pretreatment with PP2 (Figure 6A–H). Increased p-Fyn and p-Src expression in response to LPS were also confirmed by immunofluorescence staining, and these levels were decreased by the pretreatment with PP2 (Figure 6I).

### 2.6. PP2 Attenuates Mitochondrial Dysfunction in LPS-Induced AKI

**Figure 7:**
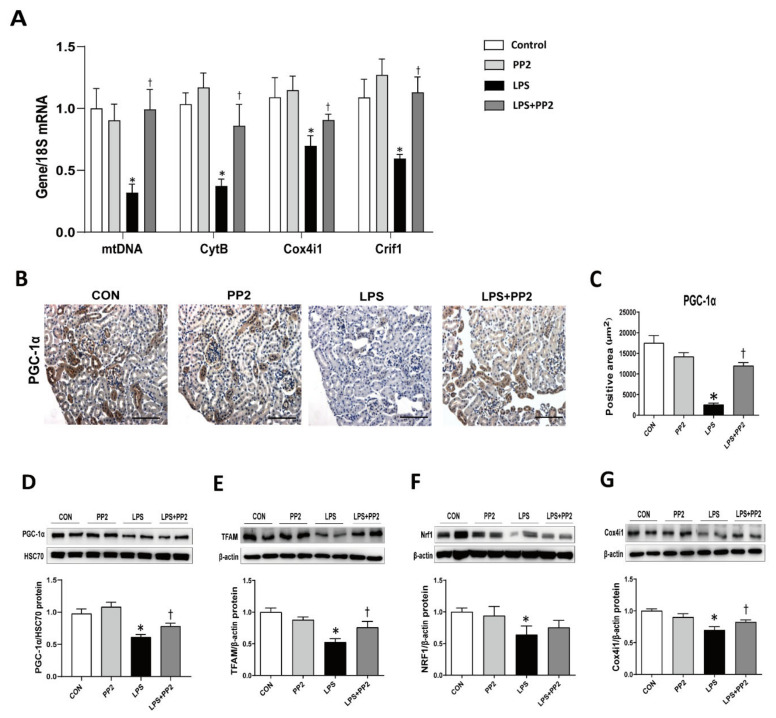
PP2 preserves mitochondrial biogenesis in LPS-induced AKI mice. (**A**–**G**) Six-week-old male C57/BL6 mice were pretreated with PP2 (2 mg/kg) for 2 h and then administered LPS (15 mg/kg) for 18 h. (**A**) The mRNA levels of mtDNA, CytB, Cox4i1, and Crif1 in kidney tissue were measured using real time RT-PCR analysis. The levels of mRNA were normalized with 18S. (**B**–**C**) Paraffin-embedded kidney sections were stained with PGC-1α antibody (1:200; original magnification: 200×; scale bar: 100 μm). Immunoblotting analysis of (**D**) PGC-1α, (**E**) TFAM, (**F**) Nrf1, and (**G**) Cox4i1 in the kidney was performed. The levels of proteins were normalized with HSC70 and β-actin. Data are presented as mean ±SE of 6–8 mice * *p* < 0.05 vs. control, ^†^ *p* < 0.05 vs. LPS.

We measured the expression of mitochondrial fitness markers in the kidneys of LPS-induced AKI mice. LPS reduced the mRNA expression levels of mtDNA, CytB, Cox4i1, and Crif1 in AKI mice, which were all prevented by PP2 (Figure 7A). In addition, the protein expression of PGC-1α TFAM, Nrf1, and Cox4i1 were also reduced in LPS-induced AKI mice (Figure 7B–G). Pretreatment with PP2 effectively restored LPS-induced suppression of PGC-1α TFAM, Nrf1, and Cox4i1 protein expression in the kidneys of AKI mice (Figure 7B–G).

## 3. Discussion

In the present study, we demonstrated that (i) inhibition of SFK ameliorated LPS-induced kidney injury and mitochondrial dysfunction, and (ii) the expression and activation of Fyn and Src were increased in LPS-induced AKI.

SFK have been discovered to play important roles in cancer biology including the regulation of cell growth, differentiation, metabolism, and apoptosis [10]. In addition, SFK have long been recognized to participate in pathological conditions related to inflammatory responses [31]. Recent studies have also suggested a possible role of SFK in kidney diseases [14,15,16,18,32].

The sepsis-induced AKI is characterized by parenchymal inflammation, endothelial dysfunction, intra-glomerular thrombosis, and tubular injury [5,6,7]. The endothelium is the primary target of endotoxin, and endothelial dysfunction is considered as a key player leading to exacerbated inflammatory response and kidney failure [6,7]. In addition, LPS induced pathological ultrastructural changes of mitochondria in both renal tubular epithelial cells and the endothelial cells, and mitochondrial-targeted antioxidants protected LPS-induced AKI [33].

We first confirmed that LPS induces kidney dysfunction, estimated by plasma creatinine and cystatin C, and kidney injury, including inflammation and oxidative stress, in the proximal tubular epithelial cells. In agreement with previous studies that showed mitochondrial dysfunction in AKI [23,24,26], mRNA expression of PGC-1α as well as other markers of mitochondrial fitness including TFMA, Nrf1, mtDNA, and CytB were decreased in response to LPS. PGC-1α is an important regulator of mitochondrial biogenesis in organs with high energy demands, including the kidney [25]. Importantly, pretreatment with PP2 effectively attenuated LPS-induced suppression of mitochondrial biogenesis markers in mProx cells and AKI mice, as well as decreasing the level of mitochondria staining by MitoTracker in mProx cells.

In line with our data, overexpression of Src impaired mitochondrial energy metabolism via regulating OXPHOS in HepG cells. The decrease in the OXPHOS complex was, as expected, increased by PP2 [30]. SFK are not only constitutively expressed in the mitochondria [27] but can also be translocated into the mitochondria from the cytoplasm, a process that is dependent on EGFR [34], AKAP121 [35], and Dok-4 [36]. Although the pharmacologic inhibition of SFK improved mitochondrial biogenesis in LPS-induced AKI, the detailed mechanisms and functional link between SFK and dysregulation of mitochondrial biogenesis remains to be studied.

Pretreatment with PP2 inhibited the increases in plasma creatinine and cystatin C normally found in LPS-induced AKI mice. In addition, PP2 pretreatment effectively protected against LPS-induced kidney inflammation and oxidative stress. Inflammation is an important component in the establishment of AKI [37]. Infiltrating macrophages exacerbate kidney injury by producing high levels of cytokines such as TNF-α and ICAM-1 [38]. Similar to our observations, the genetic modification and pharmacological inhibition of SFK attenuated inflammatory responses in ischemic brain injury [39] and acute lung injury [40].

SFK are activated under oxidative stress [41], and the cysteine residues of SFK are redox sensitive [42]. Oxidation of the residues such as Cys245 and Cys487 causes a conformational change that contributes to the activation of SFK [42]. Conversely, inhibition of Src by PP2 attenuates hyperoxia-mediated ROS through the inhibition of NADPH oxidase [43]. These studies suggest that SFK are activated by oxidative stress through direct oxidation of structural domains [42] and SFK may also act upstream of NADPH oxidase, thereby contributing to increased oxidative stress [43].

Although limited, previous studies have shown that Src was activated in I/R-induced AKI [18,44], while Fyn was increased in UUO-induced CKD [15]. Among the eight family members, only the levels of Fyn and Src expression were elevated as early as 6 h after LPS administration to mice and mProx cells. LPS-induced activation of Fyn and Src were inhibited by pretreatment with PP2. Interestingly, our study showed that not only phosphorylation but also transcription and total protein level were increased in LPS-induced AKI, which were alleviated by PP2. Although PP2 decreased the transcription and total protein level under our experimental conditions, the detailed mechanisms involved are not clear.

Several questions related to our findings remain to be answered. First, considering that Src activation is an immediate downstream target of TLR4 activation [45,46], the preservation of mitochondrial biogenesis could be a result of decreasing LPS signaling rather than a direct effect of the Src inhibition. To clearly show the functional link between SFK and mitochondria biogenesis, it is necessary to perform experiments on kidneys with selective overexpression of SFK as well as mitochondrial biogenesis molecules. Second, considering the clinical implication, further studies on therapeutic effects of PP2 in AKI are needed. Third, considering the role of endothelial damage in AKI, the role of SFK in the endothelial compartment warrants further study.

## 4. Materials and Methods

### 4.1. Reagents

All chemicals and reagents were purchased from Sigma-Aldrich (St. Louis, MO, USA) unless otherwise specified.

### 4.2. Cell Culture

The mProx cells were provided by Dr. Takeshi Sugaya (St. Marianna University School of Medicine, Kanagawa, Japan) and cultured as described [47]. Briefly, the cells were cultured in Dulbecco’s modified Eagle’s medium (DMEM; Invitrogen, Carlsbad, CA, USA) containing 10% bovine serum (BCS; GIBCO by Life Technologies, Carlsbad, CA, USA), 100 U/mL penicillin, 100 μg/mL streptomycin, 20 mM glucose, and 44 mM NaHCO_3_ under a 5% CO_2_ environment at 37 °C. The cells (5 × 10^5^ cells/well) were seeded in 6-well plates. Sub-confluent cells were allowed to growth-arrest with DMEM containing 1% BCS for 18 h before starting the experiments. The cells were pretreated with PP2 (0, 1, 5, and 10 μM, dissolved in dimethyl sulfoxide (DMSO)) for 2 h and then stimulated with LPS (100 ng/mL, dissolved in distilled water) for 9 h.

### 4.3. Animals

All experimental animals were approved by the Institutional Animal Care and Use Committee at Ewha Womans University (Approval number: IACUC No. 16-055, 12 October 2016). Six-week-old male C57BL/6 mice (Japan SLC Inc., Hamamatsu, Japan) were used. Mice were randomly divided into four groups: (i) control, (ii) LPS 6 h, (iii) LPS 12 h, and (iv) LPS 24 h. Mice were examined for the induction of AKI at 6, 12, and 24 h using a single intraperitoneal (i.p.) injection of LPS (15 mg/kg).

In another set of experiments, mice were also divided into four groups: (i) control, (ii) PP2, (iii) LPS, and (iv) LPS treated with PP2. AKI induction was confirmed by single i.p. injection of LPS (15 mg/kg) for 18 h. Control mice were administrated with an equivalent volume of saline as the vehicle of LPS. PP2 (2 mg/kg) was administered (i.p.) to the mice 2 h before the injection (i.p.) of LPS, and control mice were injected with an equivalent volume of DMSO as the vehicle of PP2. All mice were sacrificed at the above time points after injection of LPS.

### 4.4. Measurements of Blood Parameters

Blood was centrifuged at 3000 rpm for 15 min at 4 °C, and plasma was collected. Plasma creatinine (Arbor Assays, Ann Arbor, MI, USA), plasma cystatin C (MSCTC0, R&D Systems, Minneapolis, MN, USA), and plasma kidney injury molecule-1 (KIM-1, MKM100, R&D Systems) were determined by using ELISA kits according to the manufacturer’s instructions. Plasma lipid hydroperoxide (LPO) level was measured by a reaction with thiobarbituric acid as described previously [48]. The experiments were performed in duplicate.

### 4.5. Histology and Immunohistochemistry

The right kidneys were fixed with 2% paraformaldehyde-lysine-periodate, pH 7.4, dehydrated, embedded in paraffin, and sectioned. Kidneys were stained with periodic acid—Schiff (PAS) reagent. Immunohistochemistry was performed using anti-neutrophil gelatinase-associated lipocalin (anti-NGAL, 1:200; Abcam, Cambridge, MA, USA), anti-F4/80 (1:400; Santa Cruz Biotechnology, Inc., Santa Cruz, CA, USA), anti-8-hydroxy-2-deoxyguanosine (8-oxo-dG, 1:200; Trevigen, Gaithersburg, MD, USA), anti-NADPH oxidase 2 (Nox2, 1:500; Gifted by Professor Yun Soo Bae, Department of Life Science, College of Natural Sciences, Ewha Womans University, Seoul, Korea), anti-NADPH oxidase 4 (Nox4, 1:300; Gifted by Professor Yun Soo Bae), and anti-PPARγ coactivator-1α (PGC-1α, 1:200; Abcam) primary antibodies. Images were obtained by light microscopy (Carl Zeiss Microscopy, GmbH, 07745, Jena, Germany) and analyzed using ImagePro 3.5 software.

### 4.6. Immunofluorescence Staining

After deparaffinization and rehydration, kidney tissue sections were incubated with antigen retrieval solution and heated in a microwave to recover antigenicity. Non-specific binding was blocked with serum-free blocking solution for 30 min at room temperature. The sections were then incubated with anti-p-Fyn (1:100; Santa Cruz Biotechnology) and anti-p-Src (1:100; Cell Signaling Technology, Denver, MA, USA) overnight at 4 °C. Then, the sections were incubated for 1 h with Alexa 488-conjugated goat anti-mouse (1:1000; Invitrogen) or Alexa 568-conjugated goat anti rabbit (1:1000; Invitrogen) antibody. Cell nuclei were detected with 4’,6-diamidino-2-phenylindole (DAPI, 1:1000; Thermo Fisher Scientific, Waltham, MA, USA). Images were captured by a Zeiss ApoTome Axiovert 200M microscope (Carl Zeiss Microscopy).

### 4.7. Quantitative Real-Time Reverse Transcriptase Polymerase Chain Reaction (RT-PCR)

Total RNA was isolated using TRIzol reagent (Invitrogen) as described [49]. Total RNA was used to synthesize cDNA. The resulting cDNA was subjected to real-time PCR using an ABI7300 (Applied Biosystems, Carlsbad, CA, USA). Expression levels of the mRNAs were measured by real-time PCR with 20 μL reaction volume consisting of cDNA transcripts, primer pairs, SYBR Green, and PCR Master Mix (Applied Biosystems). The primer sequences are listed in Table 1. The genes were normalized using 18S as an internal control.

### 4.8. Western Blot Analysis

Whole kidney protein was extracted with lysis buffer. After centrifugation (13,000 rpm, 4 °C, 15 min), the lysate was mixed with 5× sample buffer and heated at 95 °C for 6 min. Total protein concentrations were measured using Bradford methods (BioRad Laboratories, Hercules, CA, USA). The lysate was subjected to SDS-PAGE gel electrophoresis and transferred onto a polyvinylidene difluoride (PVDF) membrane. The PVDF membranes were incubated overnight at 4 °C with the primary antibodies: anti-p-Src (1:1000; Cell Signaling Technology), anti-p-Fyn (1:1000; Santa Cruz Biotechnology), anti-PGC-1α (1:1000; Abcam), anti-TFAM (1:1000; Abcam), anti-NRF1 (1:1000; Abcam), anti-Cox4i1 (1:1000; Cusabio Biotech Co., Baltimore, MD, USA), anti-β-actin (1:1000), and anti-heat shock 70 kDa protein 8 (HSC70, 1:1000; Santa Cruz Biotechnology). The blots were reacted with peroxidase-conjugated secondary antibodies (Vector Laboratories, Inc., Burlingame, CA, USA), followed by an enhanced chemiluminescent sensitive plus reaction (BioFX Laboratories, Inc., Owings Mills, MD, USA). The positive immunoreactive protein bands were detected by LAS-3000 film (FUJIFILM Corporation, Tokyo, Japan). Each blot density was normalized to β-actin or heat shock 70 kDa protein 8 and compared with that of each control.

### 4.9. Measurement of Cellular ROS

Cellular ROS production was measured using a fluorescence dye, dihydroethidium (DHE), as described [50]. Briefly, frozen kidney sections from the AKI mice treated with or without PP2 were used. DHE (5 mM, Molecular Probes, Eugene, Oregon, USA) was applied to the frozen sections of the kidneys (5 μm) for 10 min at 37 °C to obtain the presence of ROS with red fluorescence at 561 nm followed by DAPI staining, and images were taken by using a Zeiss ApoTome Axiovert 200M microscope (Carl Zeiss Microscopy).

### 4.10. Terminal Transferase-dUTP-Nick-End Labeling (TUNEL) Assay

Apoptosis was measured using the TUNEL assay according to the manufacturer’s protocol (Roche Diagnostics, Mannheim, Germany). Briefly, after deparaffinization and rehydration, kidney tissue sections were washed with 1× phosphate-buffered saline solution (PBS) and then incubated with TUNEL reaction mixture for 60 min at 37 °C in a humidified dark chamber. The sections were subsequently washed with 1× PBS (3 times), mounted with 4’,6-diamidino-2-phenylindole, and images were taken using a Zeiss ApoTome Axiovert 200M microscope (Carl Zeiss Microscopy).

### 4.11. Measurement of Mitochondria

mProx cells were incubated with MitoTracker^®^ probes stock solution (Molecular Probes) at 1 mM for 45 min. Cells were rinsed with PBS and then incubated with 3.7% formaldehyde at 37 °C for 15 min. For permeabilization, the cells were incubated with 0.2% Triton X-100 for 10 min. Images were taken using a Zeiss ApoTome Axiovert 200M microscope (Carl Zeiss Microscopy).

### 4.12. Statistical Analyses

All results are expressed as the mean ±SE. Analysis of variance (ANOVA) was used to assess the differences between multiple groups followed by Fisher’s least significant difference (LSD) test. The level of statistical significance was set at *p* values less than 0.05.

## 5. Conclusions

**Figure 8:**
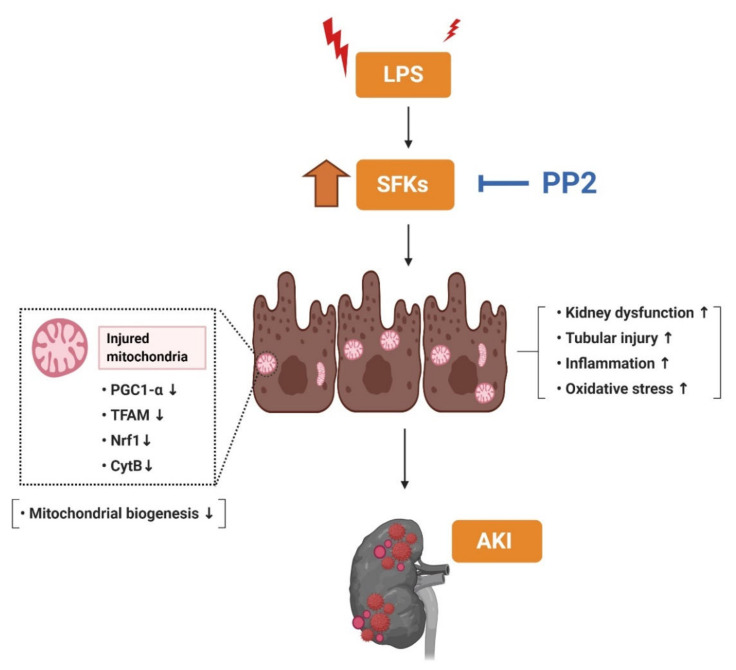
Schematic diagram illustrating that SFK activation affects mitochondrial biogenesis in LPS-induced AKI mice. The SFK inhibitor, PP2, suppressed inflammation and oxidative stress and improved mitochondrial biogenesis in the LPS-induced AKI mice.

In summary, this study demonstrated that inhibition of SFK with PP2 ameliorated dysregulation of mitochondrial fitness in LPS-induced AKI. In particular, only Fyn and Src, not other SFK, were increased in LPS-induced AKI and were decreased by PP2. Furthermore, pretreatment with PP2 ameliorated the kidney injury including the inflammation, oxidative stress, and dysregulated mitochondrial biogenesis in LPS-induced AKI. These findings suggest that inhibition of SFK, specifically Fyn and Src, can be a potential preventive strategy in AKI, possibly via preserving mitochondrial biogenesis (Figure 8).

## Figures and Tables

**Table 1:** Primer sequences used for real time RT-PCR analysis.

Gene	Forward (5′→3′)	Reverse (5′→3′)
18S	CGAAAGCATTTGCCAAGAAT	AGTCGGCATCGTTTATGGTC
Cox4i1	TCGATCGTGACTGGGTGGCCA	GCCGAGGGAGTGAGGGAGGC
Crif1	AGCTAACGCCCCGCTATGTG	ATGGTCGCTAAGCTCGGGTA
CytB	AAGAGCACCTGGGTGATCCTGCA	CGTGCATCCGTAGAGTGCCCG
Fyn	CTTTGGGGGTGTGAACTCCT	TTCTGCCTGGATGGAGTCAA
Hck	AGGGGTTAGGACTGGGAACA	CCCCAGAGATTTTGGACCCC
ICAM-1	CTTCCAGCTACCATGCCAAA	CTTCAGAGGCAGGAAACAGG
iNOS	GGCAGCCTGTGAGACCTTTG	CATTGGAAGTGAAGCGTTTCG
Lck	ACGATCTCGGGGATCATGG	GAGATCTTGCTGTCCAGTGGG
Lyn	AGCTCCAGAGGCCATCAACT	CACATCTGCGTTGGTTCTCC
mtDNA	CCACTTCATCTTACCATTTA	ATCTGCATCTGAGTTTAATC
NGAL	GGCCAGTTCACTCTGGGAAA	TGGCGAACTGGTTGTAGTCC3
NRF-1	CAACAGGGAAGAAACGGAAA	GCACCACATTCTCCAAAGGT
PGC-1α	TCGATGTGTCGCCTTCTTG	ACGAGAGCGCATCCTTTGG
Src	TCCACACCTCTCCGAAGCAA	CATGCTGATGGCCTGTGTCA
TFAM	ATTCCGAAGTGTTTTTCCAGCA	TCTGAAAGTTTTGCATCTGGGT
TNF-α	CGTCAGCCGATTTGCTATCT	CGGACTCCGCAAAGTCTAAG
Yes	TGGGAATCAGCGAGGTATTT	ACATTGTCACCCCTCACCTC

## Supplementary Materials

The following are available online at https://www.mdpi.com/1422-0067/21/21/8246/s1.
