# Supplementary material for: Inhibition of Src Family Kinases Ameliorates LPS-Induced Acute Kidney Injury and Mitochondrial Dysfunction in Mice
Source: Int J Mol Sci. 2020 Nov 3;21(21):8246. doi: 10.3390/ijms21218246 (PMC7662942; doi:10.3390/ijms21218246)
Supplement: Supplementary file 1 [file ijms-21-08246-s001.pdf]

Supplementary Materials

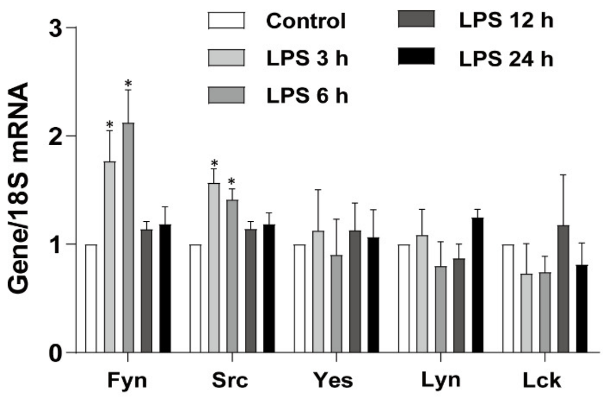

Figure S1. LPS increases expression of SFK in the mProx cells. The mProx cells were stimulated with LPS in a time-dependent manner (0, 3, 6, 12, and 24 h). The levels of SFK mRNA expression were measured using real-time RT-PCR analysis. Levels of mRNA were normalized with 18S. Data are presented as mean ± SE, $n = 4$. *$p < 0.05$ vs. control.

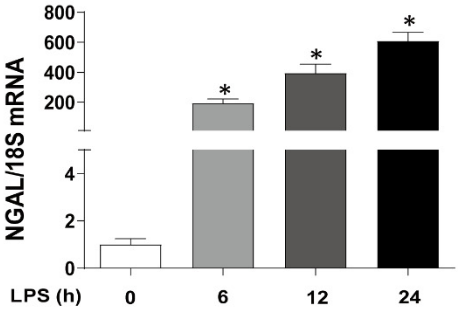

Figure S2. LPS increases mRNA expression of NGAL, a marker of tubular injury, in a time-dependent manner. Six-week-old male C57/BL6 mice were treated with LPS (15 mg/kg) in a time-dependent manner (0, 6, 12, and 24 h). The level of NGAL mRNA expression were measured using real-time RT-PCR analysis. Levels of mRNA were normalized with 18S. Data are presented as mean ± SE of 6–8 mice. *$p < 0.05$ vs. control.
